# Filtration and tubular handling of EWE‐hC3Nb1, a complement inhibitor nanobody, in wild type mice and a mouse model of proteinuric kidney disease

**DOI:** 10.1002/2211-5463.13752

**Published:** 2024-01-04

**Authors:** Morten Schøler Fast, Kathrin Weyer, Henrik Pedersen, Gregers Rom Andersen, Henrik Birn

**Affiliations:** ^1^ Department of Biomedicine Aarhus University Denmark; ^2^ Department of Molecular Biology and Genetics – Protein Science Aarhus University Denmark; ^3^ Departments of Clinical Medicine Aarhus University and Renal Medicine, Aarhus University Hospital Denmark

**Keywords:** complement, EWE‐hC3Nb1, megalin, nanobody, pharmacokinetics, proteinuria

## Abstract

Tubular activation and deposition of filtered complement proteins have been implicated in the progression of proteinuric kidney disease. The potent C3b‐specific nanobody inhibitor of the alternative pathway, EWE‐hC3Nb1, is likely freely filtered in the glomerulus to allow complement inhibition in the tubular lumen and may provide a novel treatment option to prevent tubulointerstitial injury. However, more information on the pharmacokinetic properties and renal tubular handling of EWE‐hC3Nb1 nanobody is required for its pharmacological application in relation to kidney disease. Here, we examined the pharmacokinetic properties of free EWE‐hC3Nb1 in mouse plasma and urine, following subcutaneous injection in wild‐type control and podocin knock out (KO) mice with severe proteinuria. Tubular handling of filtered EWE‐hC3Nb1 was assessed by immunohistochemistry (IHC) on kidney tissue from control, proteinuric mice, and KO mice deficient in the proximal tubule endocytic receptor megalin. Rapid plasma absorption and elimination of EWE‐hC3Nb1 was observed in both control and proteinuric mice; however, urinary excretion of EWE‐hC3Nb1 was markedly increased in proteinuric mice. Urinary EWE‐hC3Nb1 excretion was amplified in megalin KO mice, and substantial accumulation of EWE‐hC3Nb1 was observed in megalin‐expressing renal proximal tubules by IHC. Moreover, free EWE‐hC3Nb1 was found to be rapidly cleared from plasma. In conclusion, filtered EWE‐hC3Nb1 is reabsorbed by a megalin‐dependent process in the proximal tubules. Increased load of filtered proteins in the tubular fluid may inhibit the megalin‐dependent uptake of EWE‐hC3Nb1 in proteinuric mice. Treatment with EWE‐hC3Nb1 may allow investigation of the effects of complement inhibition in the tubular fluid.

## Graphical abstract

The potent C3b‐specific nanobody inhibitor of the alternative pathway, EWE‐hC3Nb1, is rapidly cleared by glomerular filtration from plasma following subcutaneous injection in controls and a mouse model of proteinuric kidney disease. Filtered EWE‐hC3Nb1 is reabsorbed in the proximal tubule by a megalin‐dependent process. Heavy tubular protein load, as observed in proteinuric kidney disease, may inhibit the reabsorption of EWE‐hC3Nb1 by megalin in the proximal tubule.

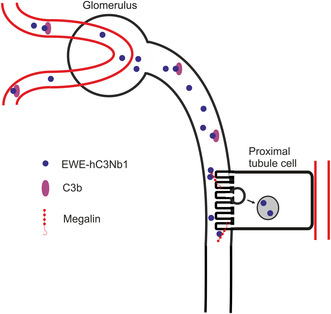

**Abbreviations**
AUC: area under the curve
BSA: bovine serum albumin
EDTA: ethylenediaminetetraacetic acid
ELISA: enzyme‐linked immunosorbent assay
GFR: glomerular filtration rate
HRP: Horseradish peroxidase
I.p.: intraperitoneal
IHC: immunohistochemistry
KO: knockout
Meg: megalin
Nph2: podocin
PBS: phosphate‐buffered saline
RT: room temperature
S.c.: subcutaneous
SD: standard deviation
TBS: tris‐buffered saline
TBST: TBS containing 0.05% Tween 20
TMB: 3,3′,5,5′ tetramethylbenzidine

Glomerular proteinuria is associated with progressive kidney disease, tubular injury, and interstitial fibrosis [1, 2]; however, the disease mechanisms remain largely unknown. Activation of the complement system through the alternative pathway has been hypothesized to play a key role in the progression of proteinuric kidney disease [3]. The alternative complement pathway is constitutively active at low levels through the body [4], and regulation by plasma and membrane‐bound regulatory proteins is necessary to prevent injury to healthy host cells [5, 6]. Insufficient regulation of the complement system may lead to tissue injury and inflammation [7, 8]. Renal tubular cells show scarce expression of complement inhibitors on the brush border, and the proximal tubule has been hypothesized to be vulnerable to attacks from complement proteins in the luminal tubular fluid [9]. Inhibition of complement activation in the renal tubular fluid may provide a novel treatment of progressive proteinuric kidney disease.

Disruption of the glomerular filtration barrier in the kidney causes proteinuria due to leakage of plasma proteins, including complement proteins, into the glomerular filtrate and tubular fluid [10]. The brush border of renal proximal tubule cells is capable of complement activation through the alternative pathway [11, 12], and inhibitors of complement activation targeted to the proximal tubule have been shown to prevent tubulointerstitial injury in an experimental rat model of nephrotic syndrome [13]. Increased levels of complement activation products have been observed in urine from patients with glomerular diseases [10], and biopsies from diseased human kidneys have shown accumulation of complement activation products in areas of tubulointerstitial damage [14, 15]. Complement activation in the tubular fluid by filtered complement factors seems to be an important mediator of tubular injury in glomerular proteinuric kidney diseases [2, 3] and may cause progression of various proteinuric kidney diseases such as autoimmune glomerular disease, ischemia–reperfusion injury, preeclampsia, and diabetic nephropathy [8, 16, 17, 18].

EWE‐hC3Nb1 is a nanobody‐based complement inhibitor, which inhibits activation of the alternative complement pathway by binding the active complement component C3b [19, 20]. The small size and hydrophilic properties of the EWE‐hC3Nb1 nanobodies (≈15 kDa) allow free filtration across the glomerular filtration barrier to the tubular fluid [21]. EWE‐hC3Nb1 may provide a tool for investigation of the protective effects of complement inhibition in the renal tubular system; however, the pharmacological use of EWE‐hC3Nb1 requires information on its pharmacokinetic properties and renal tubular handling. The multiligand endocytic receptor megalin, expressed on the apical surface of proximal tubule cells, has previously been shown play a key role in the accumulation of nanobodies in the kidneys [22, 23] and may also be involved in the uptake of EWE‐hC3Nb1.

We aimed to examine the pharmacokinetic properties and tubular handling of free EWE‐hC3Nb1 following subcutaneous injection in controls and conditional podocin knock out mice with severe proteinuria. We also investigated if the megalin receptor is involved in uptake of EWE‐hC3Nb1 in the proximal tubules.

## Materials and methods

### Antibodies and chemicals

Bovine Serum Albumin (BSA) (Cat.No.: A7030) was purchased from Sigma‐Aldrich (St. Louis, MO, USA). HRP‐conjugated THE™ mouse anti‐HIS tag antibody (Cat.No.: A00612) was purchased from Genscript (Piscataway, NJ, USA). Sheep anti‐megalin antibody was previously described [24]. Complement C3b inhibitor nanobody, His‐tagged EWE‐hC3Nb1, was prepared as described [19]. Endotoxins were removed from the purified nanobodies as described [25]. The endotoxin levels were subsequently quantified using a ToxinSensor Chromogenic LAL Endotoxin Assay Kit (Cat.No.: L00350, Genscript). Endotoxin levels below 2 EU·mg^−1^ nanobodies were considered to be endotoxin free [26]. Human complement component C3b (Cat.No.: A114) was purchased from Complement Technology, Inc (Tyler, TX, USA).

### Free EWE‐hC3Nb1 specific ELISA

Medium bind ELISA plates (Cat.No.: 82.1581.110, Sarstedt AG & Co. KG, Nürnberg, Germany) were coated with 200 ng per well of human complement protein C3b in phosphate‐buffered saline (PBS) coating buffer and incubated overnight at 4 °C. The plates were washed four times with 200 μL tris buffered saline (TBS) containing 0.05% Tween 20 (TBST). Plates were blocked with 200 μL of TBST containing 1 mg·mL^−1^ BSA for 1 h at room temperature (RT) and then incubated for 1 h at RT with urine and plasma samples from treated mice diluted in the diluent solution consisting of TBST with 1 mg·mL^−1^ BSA and 10 mm EDTA. Plates were washed four times with TBST and subsequently incubated for 1 h at RT with HRP‐conjugated anti‐HIS‐tag antibodies diluted to 1 μg·mL^−1^ in the diluent solution. Following six final washes with TBST, the plates were incubated for 10 min at RT with 100 μL of Pierce™ TMB substrate (Cat.No.: 34021, ThermoFisher, Waltham, MA, USA). Then, 100 μL of 1 m H_2_SO_4_ was added to stop the reaction, and the absorbance was measured at 450 nm on the EnSpire Multimode Plate Reader (PerkinElmer, Waltham, MA, USA). EWE‐hC3Nb1 concentrations in the samples were determined by using a standard curve derived from serial two‐fold dilutions of EWE‐hC3Nb1 from 40 to 0.625 ng·mL^−1^ in the diluent solution, with a blank buffer control included. This produced a sigmoidal standard curve (Fig. S1). The intra‐ and inter‐assay coefficients of variation were 5.91% and 8.57%, respectively, in plasma, and 7.43% and 6.74%, respectively, in urine (Figs S2 and S3); thus, the coefficients of variation were low at less than 10% both intra‐ and inter‐assay, in both plasma and urine. As the ELISA method relies on EWE‐hC3Nb1 binding to the C3b in the wells it detects only free, his‐tagged EWE‐hC3Nb1, i.e. EWE‐hC3Nb1 that is not bound to soluble C3b.

### Animal models

Mice were maintained on 12 h light : dark cycles at a temperature of 22 °C with a humidity of 55%, and fed a standard diet. Meg^lox/lox^ and Nph2^lox/lox^ mice were crossed with tamoxifen‐inducible UBC‐cre/ERT2 transgenic mice (The Jackson Laboratory, Bar Harbor, ME, USA) [27]. Tail DNA was analyzed by PCR for genotyping. All mice were of a mixed C57BL/6‐129/Svj background. At age 8 to 12 weeks, female inducible megalin knock out (KO) (Meg^lox/lox^; Cre^+^), podocin KO (Nph2^lox/lox^; Cre^+^) and littermate control mice (Meg^lox/lox^, Nph2^lox/lox^; Cre^−^), were induced by i.p. injection of tamoxifen (Cat.No.: T5648, Sigma‐Aldrich) at a dose of 50 mg·kg^−1^ body weight for 5 consecutive days. The knockdown of the target genes in the kidney was assessed by quantitative real‐time PCR at termination, 4 weeks after the first day of tamoxifen induction. Mice with less than 70% megalin or podocin gene knock out were excluded. Mouse breeding and experiments were carried out in a certified animal facility according to provisions from the Danish Animal Experiments Inspectorate (2020‐15‐0201‐00400). Urine was collected at week 3 by housing the mice in metabolic cages for 24 h, and the urinary protein excretion was estimated by urine dipstick test (Roche Combur‐Test).

### Pharmacokinetics of EWE‐hC3Nb1 in control and podocin KO mice

Control (*n* = 10) and proteinuric podocin KO (*n* = 8) mice were injected subcutaneously (s.c.) with his‐tagged EWE‐hC3Nb1 (10 mg·kg^−1^ of body weight) in PBS. Male and female mice at 12–16 weeks were used. At *t* = 15 min, 30 min, 1 h, and 2 h; plasma was collected by tail vein blood samples. At *t* = 3, 6, and 10 h plasma and bladder urine samples were collected from anesthetized mice that were euthanized by decapitation. Tail vein blood was collected in heparinized capillary tubes while blood at termination was collected from the inferior vena cava using EDTA‐treated syringes. Kidney tissue was extracted; however, IHC performed on the tissue achieved from these was not included in this study.

### Animal experiment on the role of megalin on the urinary EWE‐hC3Nb1 excretion

Control (*n* = 5), megalin KO (*n* = 4), and podocin KO (*n* = 4) mice were injected s.c. with EWE‐hC3Nb1 (10 mg·kg^−1^ of body weight) in PBS, and the mice were places in individual clean plastic cages. Male and female mice at 12–16 weeks were used. Urines were collected by pipetting from the floor of the cage for 30 min. At *t* = 30 min, EDTA‐plasma, any remaining bladder urine, and kidney tissue were collected from anesthetized mice that were euthanized by decapitation. IHC results included in this study was all performed on the kidney tissue extracted in this way.

### Plasma, urine, and tissue samples

Urine was centrifuged at 1500 ***g*** for 3 min and stored at −80 °C. Plasma was prepared by centrifugation at 3000 ***g*** for 6 min and stored at −80 °C. The kidney was fixed by retrograde perfusion through the abdominal aorta with 2% paraformaldehyde in 0.1 mm sodium cacodylate buffer, pH 7.4. The tissue was subsequently dehydrated and embedded in paraffin by standard methods. Samples were analyzed for EWE‐hC3Nb1 nanobodies by the ELISA method described above.

### Immunohistochemistry (IHC)

Tissue preparation, sectioning, and labeling were performed as described previously [28]. IHC was performed on kidney tissue from mice euthanized 30 min after injection. Renal paraffin sections were incubated with anti‐HIS‐tag antibodies overnight at 4 °C. Antibody labeling was visualized by incubation with diaminobenzidine and 0.03% H_2_O_2_ for 10 min, and sections were counterstained with Mayer's hematoxylin (Sigma‐Aldrich). Images were acquired with a Leica DMR microscope equipped with a Leica DFC320 camera (Leica, Wetzlar, Germany). All microscope and camera settings were kept identical when obtaining images from different groups.

### Statistical analysis

The total amount of EWE‐hC3Nb1 excreted in the urine from control and megalin KO mice after 30 min was calculated based on the collected, total urine volume. EWE‐hC3Nb1 concentrations were expressed as the mean ± standard deviation (SD) and compared by unpaired *t*‐test with Welch's correction, using graphpad prism software. Area under the curve (AUC) was calculated using graphpad prism (GraphPad Software, Boston, MA, USA). Statistical tests were considered statistically significant if *P*‐value < 0.05.

## Results

### Plasma elimination of EWE‐hC3Nb1 is similar in control and proteinuric mice, but urinary excretion of EWE‐hC3Nb1 is significantly greater in proteinuric mice

**Fig. 1:**
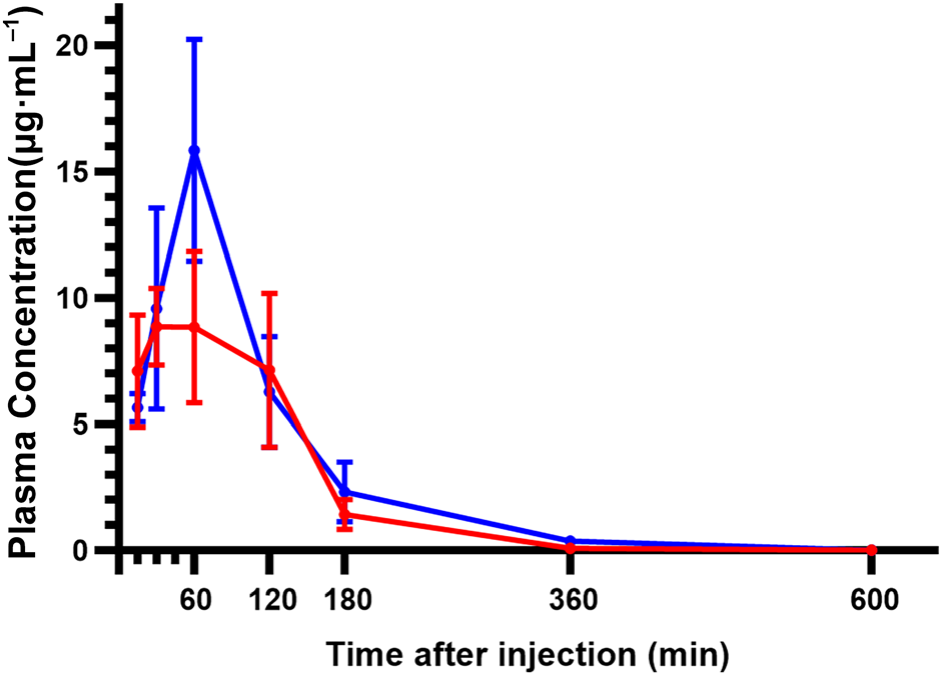
Mean EWE‐hC3Nb1 concentrations in plasma in control mice (red line) and in proteinuric mice (blue line) measured by ELISA. Mice were injected s.c. with EWE‐hC3Nb1 (10 mg·kg^−1^). EWE‐hC3Nb1 was completely eliminated from plasma 10 h after injection. The number of plasma samples used at each time point (*n*) is noted in Table 1. Error bars represent SD.

**Fig. 2:**
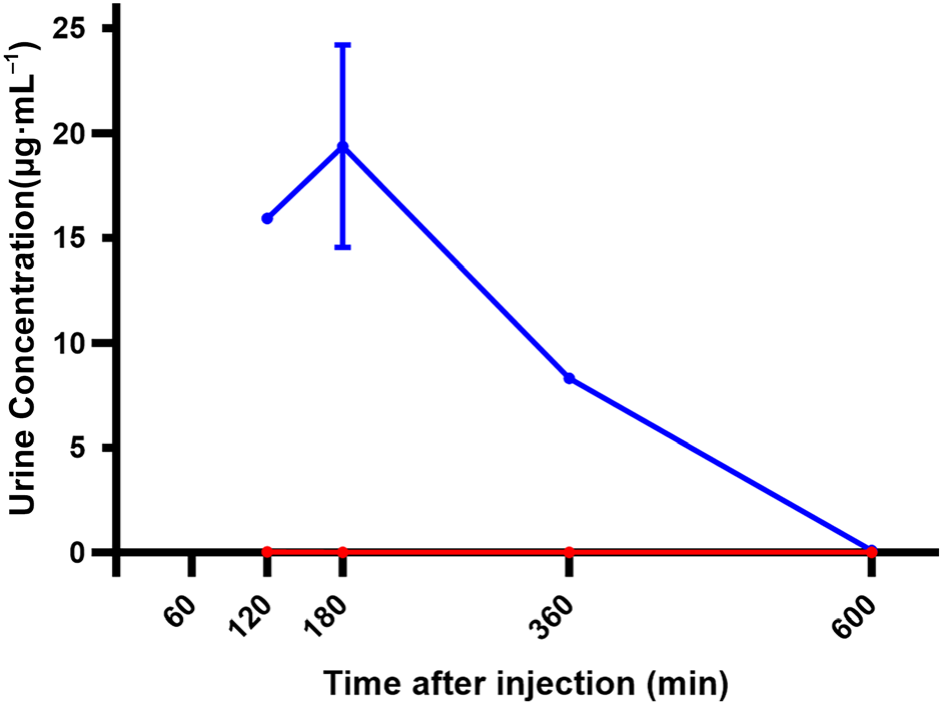
Mean EWE‐hC3Nb1 concentrations in urine in control mice (red line) and in proteinuric mice (blue line) measured by ELISA. Mice were injected s.c. with EWE‐hC3Nb1 (10 mg·kg^−1^). Urine was collected from the bladder at euthanization. Urinary EWE‐hC3Nb1 concentrations were markedly increased in proteinuric mice. The number of urine samples used at each time point (*n*) is noted in Table 1. Error bars represent SD.

The plasma concentrations of free EWE‐hC3Nb1 in control and proteinuric podocin KO mice following s.c. injection revealed an initial increase in concentration most likely reflecting nanobody absorption from the subcutaneous tissue, followed by an elimination phase (Fig. 1). Peak EWE‐hC3Nb1 concentrations in plasma occurred at *t* = 30 min to *t* = 1 h. Higher peak plasma concentrations were observed in proteinuric mice also showing a greater area under the curve (AUC) (Table 1). Urinary free EWE‐hC3Nb1 concentrations were markedly increased in proteinuric mice compared to the very low concentrations (<0.1 μg·mL^−1^) observed in control mice at all time points (Fig. 2), and the AUC in urine was significantly increased in proteinuric mice compared to control mice (*P* < 0.001) (Table 1). IHC was performed on the kidney tissue from this experiment; however, EWE‐hC3Nb1 staining was not as strong at 3 h after injection as after 30 min (data not shown) and; thus, these results were not included in this study.

**Table 1:** Concentrations of EWE‐hC3Nb1 (μg·mL^−1^) and area under curve (AUC) in plasma and urine from control and proteinuric podocin KO mice after s.c. EWE‐hC3Nb1 injection (10 mg·kg^−1^). Values are expressed as mean ± SD. The number of plasma or urine samples collected and analyzed at each distinct time point is noted in brackets (*n*=). Differences in urine nanobody AUC were statistically significant (*P* < 0.001), while differences in plasma AUC were not significant.

	Plasma		Urine	
	Control	Podocin KO	Control	Podocin KO
AUC	21.13 ± 2.93	28.36 ± 3.63	0.042 ± 0.012	75.98 ± 13.24
Time after nanobody injection				
15 min	7.10 ± 2.22 (*n* = 4)	5.66 ± 0.56 (*n* = 4)		
30 min	8.87 ± 1.52 (*n* = 6)	9.57 ± 3.99 (*n* = 4)		
1 h	8.83 ± 3.00 (*n* = 4)	15.84 ± 4.40 (*n* = 4)		
2 h	7.14 ± 3.05 (*n* = 5)	6.28 ± 2.20 (*n* = 2)	0.042 ± 0.004 (*n* = 2)	15.93^a^ (*n* = 1)
3 h	1.43 ± 0.59 (*n* = 4)	2.32 ± 1.18 (*n* = 3)	0.011 ± 0.008 (*n* = 4)	19.39 ± 8.37 (*n* = 3)
6 h	0.085^a^ (*n* = 1)	0.36^a^ (*n* = 1)	0.000^a^ (*n* = 1)	8.30^a^ (*n* = 1)
10 h	0.000^a^ (*n* = 1)	0.003^a^ (*n* = 1)	0.000^a^ (*n* = 1)	0.96^a^ (*n* = 1)

^a^ No SD was calculated due to *n* = 1 at these time points.

### EWE‐hC3Nb1 accumulates in renal proximal tubule cells following injection

**Fig. 3:**
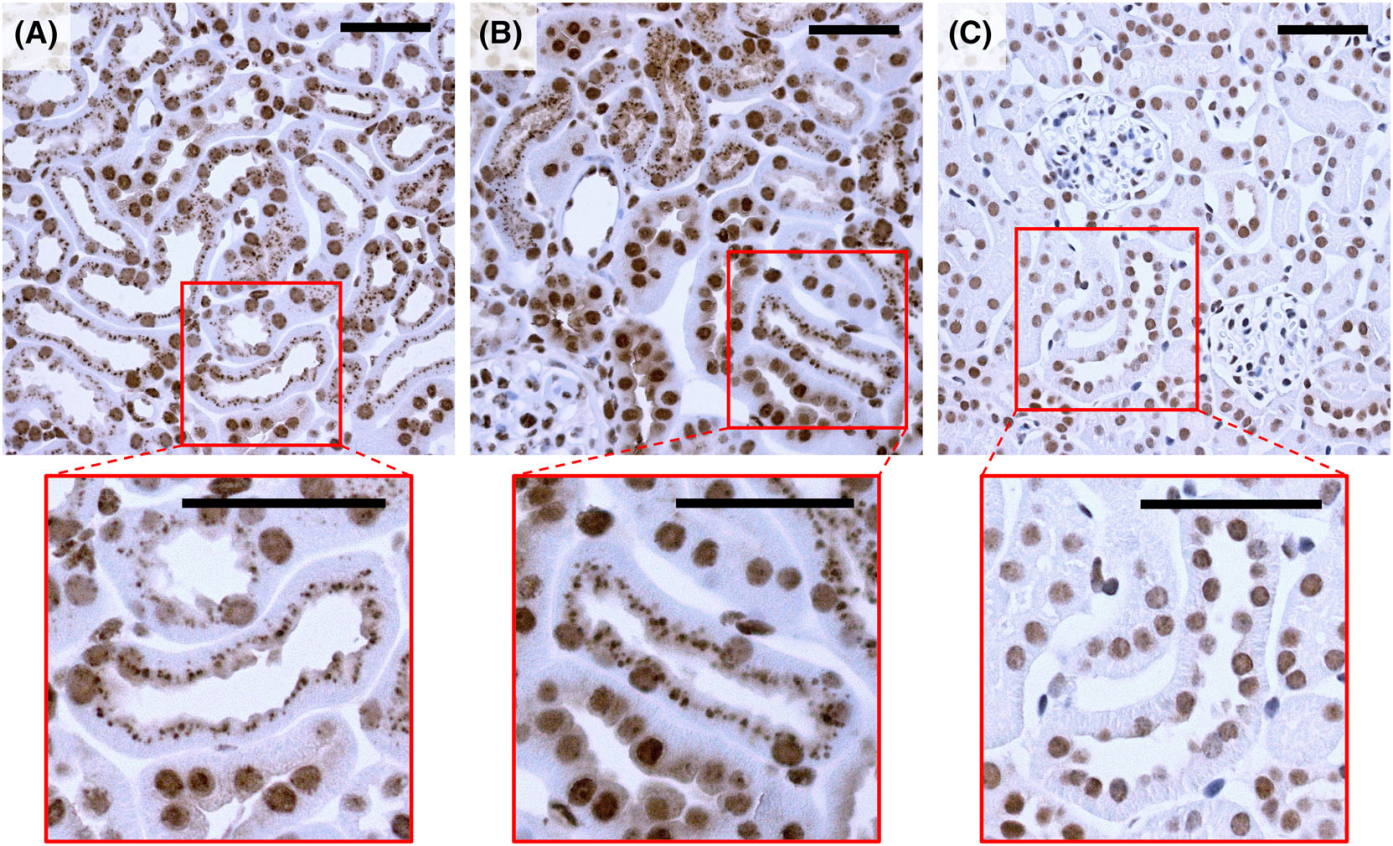
Tubular accumulation of EWE‐hC3Nb1 30 min after injection. Kidney tissue from control mice (A) and proteinuric podocin KO mice (B) at 30 min after his‐tagged EWE‐hC3Nb1 injection were stained for EWE‐hC3Nb1 with anti‐his antibody. Staining for EWE‐hC3Nb1 was observed in the luminal membrane and cytosolic vesicles of the proximal tubules. Kidney tissue from untreated control mice (C) demonstrates the nuclear background staining. Scale bars 50 μm.

The renal uptake of EWE‐hC3Nb1 nanobodies was explored by performing IHC on kidney tissue from both control and proteinuric podocin KO mice at 30 min after EWE‐hC3Nb1 injection. EWE‐hC3Nb1 staining was observed in the luminal membranes and apical vesicles in proximal tubules of both control (Fig. 3A) and podocin KO mice (Fig. 3B). A background, nuclear staining was observed in all kidney tissues with the anti‐his antibody, including kidney from untreated mice (Fig. 3C).

### Accumulation of EWE‐hC3Nb1 in tubular cells relies on apical megalin expression

**Fig. 4:**
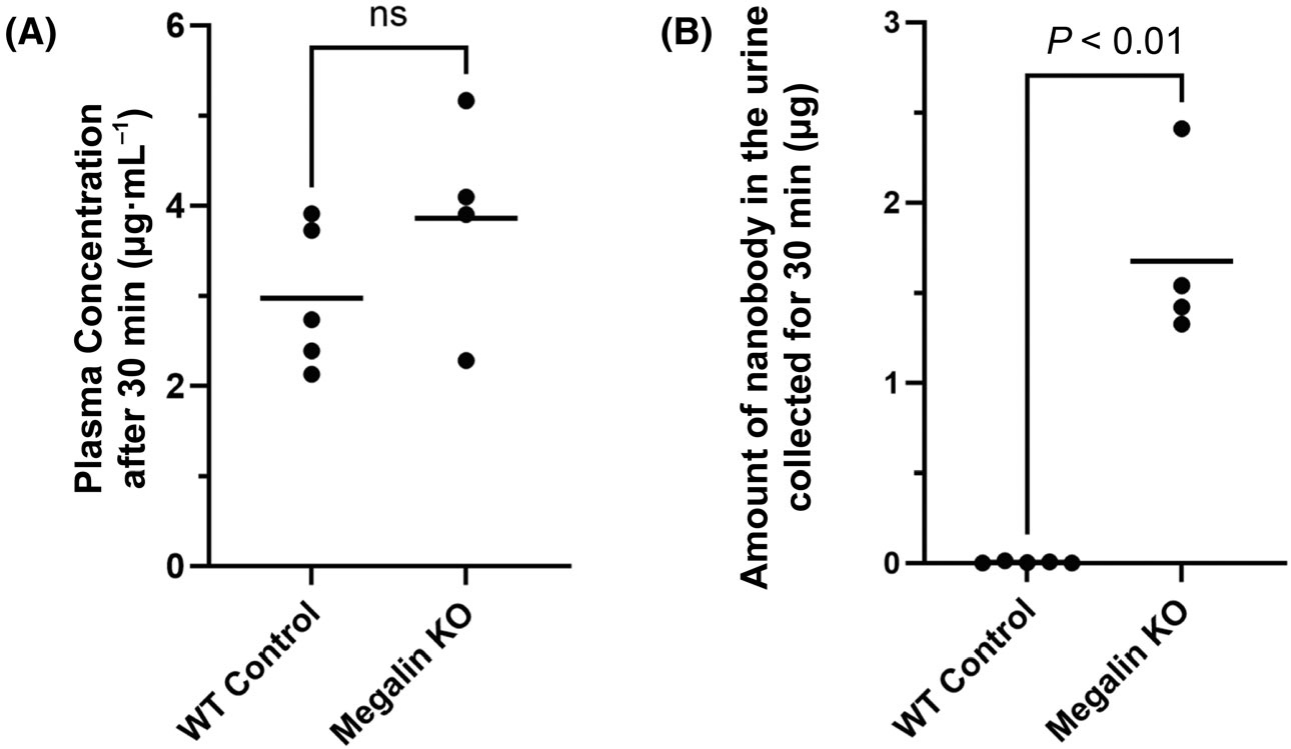
Urinary EWE‐hC3Nb1 excretion in controls (*n* = 5) and megalin‐deficient mice (*n* = 4) 30 min after injection. Mice were injected s.c. with EWE‐hC3Nb1 (10 mg·kg^−1^), and urine was collected continuously for 30 min. Plasma and the remaining urine were collected 30 min after injection at euthanization. EWE‐hC3Nb1 concentrations in control and megalin KO mice in plasma 30 min after injection (A), and the total amount of nanobody (μg) in the urine collected for 30 min (B). The amount of nanobody in the urine was marked increased in megalin KO mice. The data was compared using an unpaired *t*‐test with Welch correction (ns, Not significant).

**Fig. 5:**
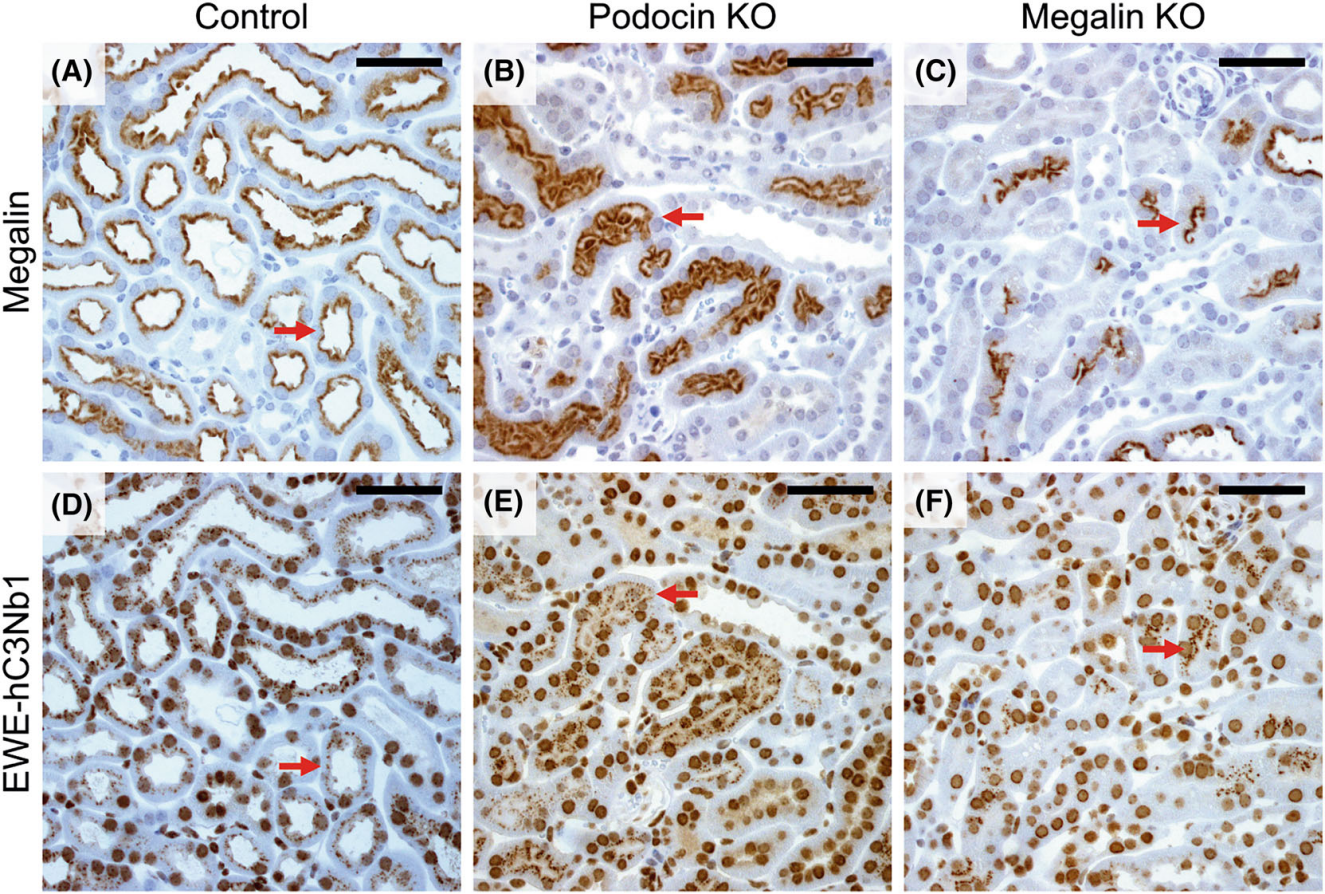
Tubular megalin expression and EWE‐hC3Nb1 reabsorption 30 min after injection. Serial sections of kidney tissue from control mice (A, D), proteinuric podocin KO mice (B, E) and megalin KO mice (C, F) were stained for megalin (A–C) or EWE‐hC3Nb1 (D–F). Megalin expression is present in the proximal tubules of controls (A) and proteinuric podocin KO mice (B). Tubular expression of megalin is reduced with remnant mosaic expression in the megalin knock out mouse model (C). Areas with accumulation of EWE‐hC3Nb1 corresponds with areas of proximal tubule cells with intact megalin expression in controls, proteinuric podocin KO mice, and megalin KO mice (red arrows). Scale bars 50 μm.

Using megalin KO mice, we next investigated if the megalin receptor is involved in the renal uptake of EWE‐hC3Nb1 nanobodies. Free EWE‐hC3Nb1 plasma concentrations were similar in control and megalin KO mice at 30 min after s.c. EWE‐hC3Nb1 injection; however, free urinary EWE‐hC3Nb1 excretion was markedly increased in megalin KO mice (Fig. 4). IHC on serial sections of kidney tissue from megalin KO mice at 30 min after injection of EWE‐hC3Nb1 showed reduced tubular EWE‐hC3Nb1 staining with patchy labeling in some tubules, consistent with the residual expression of megalin observed in a few tubules in megalin‐deficient mice (Fig. 5). Megalin staining seems to be stronger at the brush border of podocin KO mice compared to the control mice, however, previous studies from our lab have not shown any increase in megalin expression [28]. The variation in staining intensity may be due to slight variations in the staining protocol.

## Discussion

We examined the pharmacokinetic properties following single subcutaneous injection of EWE‐hC3Nb1 in control and proteinuric mice. Free EWE‐hC3Nb1 is rapidly cleared from the circulation involving renal glomerular filtration followed by megalin‐mediated uptake in the proximal tubules. In megalin‐deficient mice and in proteinuric mice with intact megalin expression, free EWE‐hC3Nb1 was identified at high levels in the urine.

The free EWE‐hC3Nb1 nanobody is hydrophilic and of small size (≈15 kDa) consistent with a significant first‐pass clearance through the kidneys by glomerular filtration and consequently rapid elimination from plasma. This is supported by the increased urinary EWE‐hC3Nb1 excretion observed in megalin‐deficient mice with defective tubular protein reabsorption and no glomerular dysfunction. Despite the rapid clearance from plasma, only minute amounts of free EWE‐hC3Nb1 were excreted in the urine from control mice. The concurrent accumulation of nanobody in apical vesicles of kidney proximal tubular cells from control mice suggests that the nanobody is effectively reabsorbed. The megalin receptor has previously been shown to play a key role in the reabsorption of other nanobodies by endocytosis and trafficking to lysosomal vesicles for degradation in the proximal tubule [23]. The significant increase in urinary excretion and the reduced accumulation of EWE‐hC3Nb1 in tubular cells observed in megalin‐deficient mice confirms the role of megalin in nanobody reabsorption in the proximal tubule. In addition to megalin‐mediated uptake, binding of EWE‐hC3Nb1 to deposited C3b on tubular cells may contribute to the accumulation of nanobody in the proximal tubule, as deposition of complement proteins have previously been observed on the luminal surface of proximal tubules in proteinuric kidneys [3, 29, 30].

Urinary excretion of free EWE‐hC3Nb1 was increased in the proteinuric podocin KO mice with intact megalin expression on the brush border when compared to control. As discussed above EWE‐hC3Nb1 is most likely freely filtered even in the normal glomerulus and unlikely to be affected by increased glomerular permeability to proteins. This notion is supported by the fact that EWE‐hC3Nb1 plasma concentrations were not reduced in the proteinuric podocin KO mice. Thus, impairment of the tubular reuptake mechanisms in the proteinuric podocin KO mice must be implicated in the increased urinary EWE‐hC3Nb1 excretion. Glomerular proteinuria leads to substantial amounts of plasma proteins being filtered into the tubular fluid, in particular albumin, which may reduce megalin‐mediated uptake of freely filtered proteins through competitive inhibition. Previous studies have observed increased urinary excretion of other low molecular weight proteins in glomerular disease [31].

Slightly higher peak nanobody concentration and increased AUC were observed in plasma from proteinuric podocin KO mice, which may be secondary to either increased nanobody absorption following s.c. injection or reduced nanobody elimination. Increased serum creatinine as a marker of reduced GFR has previously been observed in podocin knockout mice 6 weeks after tamoxifen gene induction [32]. Although in this study the mice were injected with nanobodies 4 weeks after tamoxifen gene induction a reduced GFR cannot be excluded.

Previous micropuncture studies have shown near‐equal concentrations of low molecular weight proteins in the glomerular filtrate and plasma, with decreasing concentrations throughout the nephron [33]. Based on this, the free EWE‐hC3Nb1 concentrations in the ultrafiltrate following s.c. injection in mice is estimated at greater than 1 μg·mL^−1^ for at least 4 h. Reduced nanobody reabsorption from the tubular fluid as observed in proteinuric mice may result in higher nanobody concentrations further down through the nephron. However, it remains unknown if the nanobody concentration achieved in the tubular fluid is sufficient to obtain a measurable effect on intratubular complement activation. Larger nanobody concentrations in mice may be achieved for a shorter duration by intraperitoneal or intravenous bolus injections of the same dose (10 mg·kg^−1^). Constant plasma concentrations of EWE‐hC3Nb1, allowing constant perfusion of the proximal tubule with nanobodies, may be achieved by nanobody administration through surgically implanted osmotic pumps.

In conclusion, both in normal as well as proteinuric mice free EWE‐hC3Nb1 is rapidly cleared from plasma after subcutaneous injection. Filtered nanobody is reabsorbed by a megalin‐dependent process. In proteinuric mice, free EWE‐hC3Nb1 can be detected in urine possibly due to inhibition of megalin‐mediated uptake by the increased load of filtered proteins. Treatment with EWE‐hC3Nb1 with the aim to inhibit tubular complement activation appears feasible, but may require slower and prolonged administration. Future studies should investigate the effects of this on intratubular complement inhibition and the progression of kidney disease.

## Conflict of interest

Henrik Birn has received a research grant from Glaxo Smith Kline and Vifor Pharma (paid to institution) and has received consultancy fees and/or speaker honorarium from Vifor Pharma, AstraZeneca, Galapagos, Alexion, MSD and Novo Nordisk, as well as support for attending meetings by Novartis and AstraZeneca. He serves as the President of the Danish Society of Nephrology and a member of a working group established by the Danish Health Authority and the Danish Society of Nephrology.

### Peer review

The peer review history for this article is available at https://www.webofscience.com/api/gateway/wos/peer‐review/10.1002/2211‐5463.13752.

## Author contributions

Conceptualization, HB, KW, GRA, and MSF; methodology, HB, KW, and MSF; formal analysis, HB, KW, and MSF; investigation, MSF; resources, KW, HP, GRA, and MSF; data curation, HB, KW, MSF; writing – original draft, MSF; writing – review & editing, HB, KW, GRA, MSF; visualization, MSF; supervision, HB, KW, and MSF; project administration, MSF; funding acquisition, HB, KW, and MSF. All authors have read and agreed to the published version of the manuscript.

## Supporting information


**Fig. S1.** Standard curve produced from the EWE‐hC3Nb1 ELISA.
**Fig. S2.** Intra‐assay variation of the EWE‐hC3Nb1 ELISA.
**Fig. S3.** Inter‐assay variation of the EWE‐hC3Nb1 ELISA.

## Data Availability

The data that support the findings of this study are available from the corresponding author; Henrik Birn (hb@clin.au.dk); upon reasonable request.
