# Supplementary material for: Filtration and tubular handling of EWE‐hC3Nb1, a complement inhibitor nanobody, in wild type mice and a mouse model of proteinuric kidney disease
Source: FEBS Open Bio. 2024 Jan 4;14(2):322–30. doi: 10.1002/2211-5463.13752 (PMC10839346; doi:10.1002/2211-5463.13752)
Supplement: Supplementary file 1 — Fig. S1. Standard curve produced from the EWE‐hC3Nb1 ELISA. Fig. S2. Intra‐assay variation of the EWE‐hC3Nb1 ELISA. Fig. S3. Inter‐assay variation of the EWE‐hC3Nb1 ELISA. [file FEB4-14-322-s001.docx]

## Supplementary figures

**Supplementary figure 1:** Standard curve from an EWE-hC3Nb1 ELISA assay as produced by the plate reader software during data interpretation. The standard curve is derived from standard concentrations ranging from 0 to 40 ng/ml of EWE-hC3Nb1, prepared as described in the Methods and Materials section. A standard curve was produced from on each individual ELISA plate from the standard concentrations to allow measurement of EWE-hC3Nb1 concentrations in samples. The nanobody concentration to response curve produced by the assay is sigmoidal.


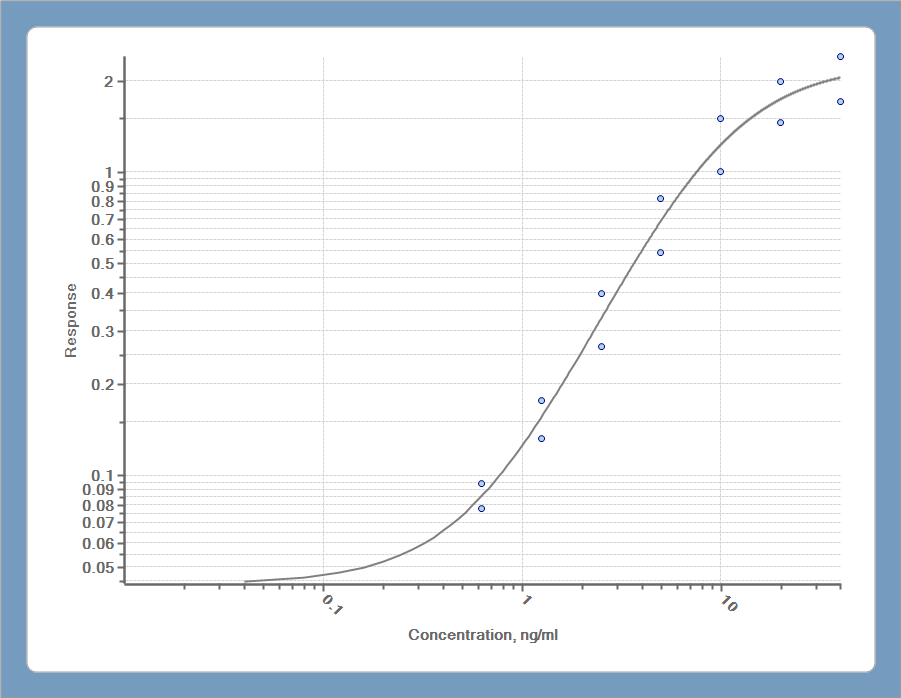


**Supplementary figure 2:** Intra-assay variation of the EWE-hC3Nb1 ELISA-assay. Plasma samples from two mice and urine samples from two mice were prepared and EWE-hC3Nb1 concentrations were measured across the same ELISA assay. Intra-assay variation was 3.91% in plasma sample #1, 7.92% in plasma sample #2, 9.16% in urine sample #1, and 5.71% in urine sample #2. Mean intra-assay variation was 5.91% in plasma and 7.43% in urine.

**Supplementary figure 3:** Inter-assay variation of the EWE-hC3Nb1 ELISA-assay. Plasma (a) and urine (b) samples from a single mouse were prepared and EWE-hC3Nb1 concentrations were measured across 5 different ELISA-assays. Inter-assay variation of the mean EWE-hC3Nb1 concentration across the 5 assays was 8.57% in plasma and 6.74% in urine.
